# Metronomic Chemotherapy in Oral Cancer: A Review

**DOI:** 10.7759/cureus.49825

**Published:** 2023-12-02

**Authors:** Sathya Sethuraman, Karthikeyan Ramalingam

**Affiliations:** 1 General Dentistry, Saveetha Dental College and Hospitals, Saveetha Institute of Medical and Technical Sciences, Saveetha University, Chennai, IND; 2 Oral Pathology and Microbiology, Saveetha Dental College and Hospitals, Saveetha Institute of Medical and Technical Sciences, Saveetha University, Chennai, IND

**Keywords:** squamous cell carcinoma, normalization, immune modulation, cytotoxic chemotherapy, anti-angiogenesis, methotrexate, celecoxib, metronomic schedule, metronomic chemotherapy, oral cancers

## Abstract

Treatment of locally advanced oral cancer requires multidisciplinary care, including surgery, radiotherapy, and chemotherapy, which varies based on the stage of the disease, site of involvement, and surgical access. Oral cancer usually presents with an increased recurrence rate and potential for distant metastatic spread. It confers a poor prognosis with a 50% mortality rate after five years.

Oral metronomic chemotherapy aims to achieve higher patient compliance due to its ease of administration, lower dosage, and lesser side effects than conventional IV regimens of platinum-based drugs. In this review, we have summarized the relevant literature to benefit the readers regarding the potential application of metronomic therapy in the management of oral cancer.

## Introduction and background

Oral cancer patients in India often present at advanced stages. Even after surgical resection and chemo-radiotherapy, 50% of such patients exhibit loco-regional recurrence and distant metastasis within 24 months [1]. Laskar et al. have reported that 60% of 900 patients showed loco-regional recurrence even after post-surgery radiotherapy [2]. Immunotherapeutic agents like nivolumab and targeted therapy like cetuximab have shown increased long-term survival but are not affordable in lower-income countries [1, 3].

Chemotherapy is very crucial in the management of recurrent or metastatic oral cancer. The gold standard regimen of platinum-based chemotherapy is cisplatin or carboplatin administration up to the maximum tolerated dose (MTD) of 100mg/m2 every three to four weeks to ensure the highest possible cancer-killing effects [4]. However, treatment with MTD is associated with systemic toxicity and has to maintain drug-free periods of three to four weeks to permit patient recovery. Genomic evolution of cancer cells can induce platinum resistance, which is also a worrisome factor [5]. 

Novel therapeutic strategies like oral metronomic therapy are being used to improve patient outcomes for several malignancies [5,6,7]. Low-dose, repetitive, regular medication delivery without extended drug-free intervals is the hallmark of metronomic therapy [5]. Yeh et al. have reported the antitumor effects of tegafur-uracil as a metronomic agent in advanced oral cancer and nasopharyngeal carcinoma [8,9]. 

## Review

Modes of action

Metronomic chemotherapy (MC) utilizes lower doses of chemotherapeutic drugs than the MTD of conventional chemotherapy. It is administered at regular intervals without any long recovery phases between cycles, thereby maintaining a consistent drug level in the blood [6,8-10].

The term metronomic is derived from metronome, a musical device that produces regular short clicks that help musicians play in rhythm. While several beneficial mechanisms of MC have been suggested, it is unknown how these mechanisms connect to each other and which mechanism is predominant for a particular tumor-drug combination [9,10].

**Figure 1:**
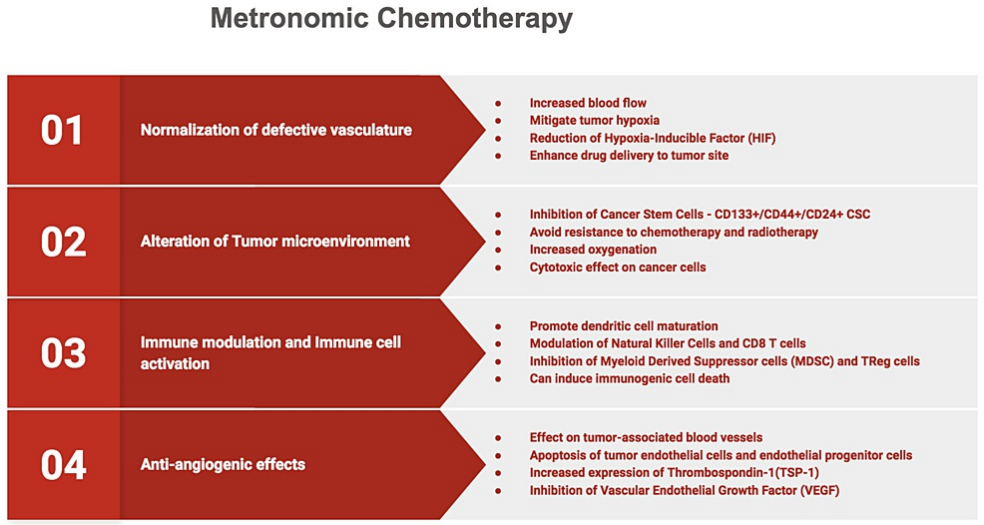
Metronomic chemotherapy Graphical representation of the action mechanisms involved in metronomic chemotherapy. Image Credit: Authors Sathya Sethuraman and Karthikeyan Ramalingam

MC can act by restricting tumor growth, obstructing tumor angiogenesis, modulating the immune system, and directly affecting tumor cells, tumor progenitors, and neighboring stromal cells. MC can have anti-angiogenic effects, vascular normalization of tumor blood vessels, immune cell activation, and reprogramming of tumor microenvironment (Figure 1) [10,11].

Clinical implications

MC has been successfully applied to a variety of malignancies. It was given as targeted therapy, vascular disrupting agents, and non-cytotoxic agents. It is either given as adjuvant or maintenance therapy immediately after curative surgery or as a mode of palliative therapy in recurrent or metastatic tumors [5]. MC is administered continuously until one of the following outcomes: surgical therapy for advanced cancer cases, clinical progression in cases with metastasis, side effects are unbearable, or fatal complications occur [6].

Two times a day, 200 mg of celecoxib administration with weekly administration of low-dose methotrexate (dosage: 15mg/m2) is the recommended protocol for MC. Celecoxib acts by inhibiting cyclo-oxygenase-2 (COX-2), which is usually overexpressed in oral cancer. Methotrexate acts by inhibiting dihydrofolate reductase leading to DNA breakage [6]. This MC offered enhanced efficiency, better tolerance, and minimal grade adverse events in comparison to cisplatin, even as palliative therapy [1]. 

In India, Pai et al. had shown that metronomic methotrexate and celecoxib during such waiting periods decreased the risk of progression and improved disease-free survival [12]. Sultania et al. showed that only 13% of locally advanced T4a tumors showed disease progression after metronomic therapy [13]. Patil et al. also showed a significant improvement in overall survival with metronomic therapy when compared with IV cisplatin [14]. But, literature also shows that pembrolizumab, cetuximab, lapatinib, afatinib, and avelumab did not improve disease-free survival [1,6].

Ramalingam et al. have reported that overall health score and quality of life score among patients with advanced tumors consistently declined with time, irrespective of the provided treatment (surgery or radiotherapy) [15]. Despite advances, oral cancer patients have a five-year mortality rate of about 50%. Advances in early diagnosis and more personalized therapy to minimize morbidity are being investigated in oral cancer patients. Circulating exosomes and genetic mutations could shed some light on the intricate pathogenesis of oral cancer and help in deciphering appropriate treatment protocols (Table 1) [16-18].

**Table 1:** Comparison table for the features of metronomic and conventional chemotherapies

Metronomic Chemotherapy	Conventional Chemotherapy
Given at lower doses than the maximum tolerated dose (MTD) for a longer period	Given at the MTD once in two to four weeks
Can be given continuously	Need for drug-free interval for patient recovery
Oral routes are possible and can be taken at home	Only IV administration with adequate hospital support
Increased patient compliance due to limited side effects	Reduced patient compliance due to toxic side effects
Plasma drug concentration is maintained consistently	Rise and fall of drug concentrations in the plasma based on cycle
Cost-effective	Expensive
Targets tumor vasculature and endothelial cells	Targets proliferating tumor cells

Li et al. have performed a systematic review and meta-analysis of MC for head and neck cancers. The outcome was negative but with limited adverse effects. The treatment duration was extended with a lower dosage, but its efficiency should be confirmed with randomized control trials [19].

Several patients with advanced cancer would not pursue further chemotherapy unless their treatment has tolerable side effects with maintenance of the functional and psychological quality of life [19,20]. Conventional chemotherapy with a combination of two or three drugs is administered for four to six cycles but has to be restricted due to toxic side effects [20]. The functional and psychological quality of life should be maintained for cancer patients [19]. 

Dose-dense chemotherapy is based on the Norton-Simon model. It uses fractionated doses to target rapidly growing small tumors and attempts to reduce co-morbidity. There are no variations in the drug dosage, but the gap between cycles is reduced. It is effective for breast carcinomas, ovarian carcinomas, and high-risk lymphomas [21]. Cem et al. have also reported in their systematic review that metronomic methotrexate and celecoxib started pre-operatively and continued as maintenance of standard treatment protocol showed better disease-free survival than standard chemotherapy [22]. 

Cost is one of the important factors in health economics and decision-making when assessing the cost-effectiveness of a drug [6]. Oral metronomic therapy is an affordable treatment option with low-cost, oral administration and does not need cold-chain maintenance. It also offers comparable disease control and a favorable toxicity profile to systemic chemotherapy. It reduces the overall healthcare cost and can be used to treat patients in remote areas [7]. MC has been endorsed for its low cost and increased patient compliance, especially for cases with progressive cancers involving the head and neck (Table 2) [19].

**Table 2:** Drugs, route of administration, and the frequency of drugs used in metronomic chemotherapy

Drugs Used in Metronomic Chemotherapy	Route	Dosage
Cyclophosphamide	Oral	Daily
Methotrexate	Oral	Twice a week
Thalidomide	Oral	Daily
Dexamethasone	Oral	Daily
Celecoxib	Oral	Twice a day
Prednisone	Oral	Daily

Limitations of MC and its potential role in India

The limitations of MC are as follows: oral metronomic regimen can only be used for patients without any swallowing impairment, and there is questionable patient compliance over the long treatment duration of one to two years [22].

As per the National Cancer Registry Program 2020, oral cancer makes up one-third of head and neck cancers. Males and females showed a higher incidence of oral cancers. A total of 38.2% of females were illiterate compared to 18.7% of males. In total, 36012 cases of oral cancer were reported in men and 11098 cases in females. The locoregional spread was noted in more than 70% of oral cancer patients. A higher incidence of tongue cancer was noted among both sexes in Nagpur, Delhi, Chennai, and Bangalore. Tongue cancer incidence was the highest in the world with 12.8 per 100000 males in East Khasi Hills district and 4.0 per 100000 females in Bhopal. Multi-modality treatment is preferred for cancers with loco-regional and distant spread comprising surgery, radiotherapy, and systemic chemotherapy [23].

It is very difficult to establish the appropriate low dose for an individual, as a balance should be maintained between therapeutic efficacy and patient tolerance. Shaked et al. have reported the estimation of circulating endothelial precursor cells as a surrogate marker to evaluate the actual efficiency of MC [24].

Optimal dosage of the metronomic regimen with new drug combinations is yet to be standardized with randomized phase III trials [22]. Frequently noted side effects are fatigue, nausea, vomiting, anemia, and a decrease in WBCs. Long-term administration of MC can lead to cumulative toxicity, especially in children and adolescents [25]. Studies have reported that endothelial cells could show anti-coagulation events, vascular co-option of normal vasculature, evasive resistance to vascular endothelial growth factor (VEGF) pathway inhibitors, vascular remodeling, and intrinsic tolerance due to changes in tumor micro-environment that ultimately disrupts the anti-angiogenic effect of MC. Tumors can show continuous proliferation in spite of hypoxia and lack of nutrition [26,27]. 

Overcoming drug resistance is a critical factor in clinical cancer research. Pharmacogenetics of MC should be deciphered for various tumors. Gene expression profiling should be performed on resistant tumors to identify the appropriate target drug for MC [27]. Prolonged waiting time can be a serious factor in head and neck cancer patients, as 30% can show progressive disease and 16% can show changes in tumor-node-metastasis staging [28]. Hence, alternative options should be developed for patients with advanced disease. 

Zhong et al. have reported the role of two cycles of TPF induction chemotherapy (docetaxel 75 mg/m2 on day one, cisplatin 75 mg/m2 on day one, and fluorouracil 750 mg/m2 on days one to five) followed by radical surgery and postoperative radiotherapy (54 to 66 Gy) versus up-front radical surgery and postoperative radiotherapy [29]. However, very few studies have been reported on the outcomes of metronomic therapy in oral cancer in India.

Pai et al. have tried a combination of methotrexate and celecoxib preoperatively on 32 patients with advanced operable head and neck cancers and reported a 75% response rate [12]. Nair et al. have utilized erlotinib and celecoxib in patients with operable oral cancers and reported a 60% response [28]. Sultania et al. used oral methotrexate 15 mg/m2 once a week and oral celecoxib 200 mg twice daily and reported that pre-operative MC had a 43% response rate. MC was also successfully administered for patients expecting more than a two-month delay in surgical therapy [13]. Praveen et al. have reported that a preoperative triple oral MC schedule of oral methotrexate 15 mg/m2 weekly, celecoxib 200 mg twice daily, and erlotinib 100 mg daily showed a partial response in 54% and stable disease in 34%. A total of 50% of the enrolled patients did not experience any toxicity [28]. Dhumal et al. administered triple MC with erlotinib 150 mg once daily, celecoxib 200 mg twice daily, and methotrexate weekly (phase 1 in variable dose 15-6 mg/m2 and 9 mg/m2 in phase 2) on 91 patients with platinum-refractory/early failure oral cancers. They reported 84 death events and the long-term outcome was unsatisfactory [1]. Nv et al. have used methotrexate and erlotinib with a clinically meaningful response rate of 69% [1]. Patil et al. have used oral methotrexate (15 mg/m2/week), oral celecoxib (200 mg twice daily), and erlotinib (150 mg once daily). They reported complete remission in two patients, partial remission in seven patients, stable disease in four patients, and progressive disease in two patients [1]. Harsh et al. have used oral celecoxib (200 mg twice daily) and oral methotrexate (15 mg/m2/week) and reported a positive response in 67% of patients (Table 3) [1].

**Table 3:** Metronomic therapy in India The table summarizes the drugs used and their response rate among Indian patients.

Authors	Drugs	Response Rate
Pai et al. [12]	Methotrexate, Celecoxib	75%
Nair et al. [28]	Erlotinib, Celecoxib	60%
Sultania et al. [13]	Methotrexate, Celecoxib	87%
Praveen et al. [28]	Methotrexate, Celecoxib, Erlotinib	88%
Dhumal et al. [1]	Methotrexate, Celecoxib, Erlotinib	-
Nv et al. [1]	Methotrexate, Erlotinib	69%
Patil et al. [1]	Methotrexate, Celecoxib, Erlotinib	86%
Harsh et al. [1]	Methotrexate, Celecoxib	67%

## Conclusions

Oral cancer is a major health burden in India. Most patients have limited access to surgical care and endure long waiting times for their turn of surgical intervention. Metronomic therapy has several benefits including home therapy, lesser cost, and minimal side effects. It could be utilized for patients who cannot afford conventional chemotherapy and for those patients waiting for definitive treatment. Efficient oral metronomic therapy combinations especially for the Indian population should be developed to improve patient outcomes. Long-term outcomes of double or triple MC should be assessed. Biomarkers and other evaluation tools should be developed to monitor the efficacy. The optimal therapy with maximum tumor response and the highest patient compliance will be a boon to many oral cancer patients.
